# Association Between Synchronous Occurrence of Multiple Myeloma and Carcinoma Prostate: Literature Analysis in the Context of a Case Report

**DOI:** 10.7759/cureus.48523

**Published:** 2023-11-08

**Authors:** Amitabh Kumar Upadhyay, Manoj Kumar, Abhishek Kumar, Anil Prasad, Aaditya Prakash

**Affiliations:** 1 Medical Oncology, Tata Main Hospital, Jamshedpur, IND; 2 Nuclear Medicine, Tata Main Hospital, Jamshedpur, IND; 3 Pathology, Tata Main Hospital, Jamshedpur, IND; 4 Radiation Oncology, Tata Main Hospital, Jamshedpur, IND

**Keywords:** rare, association, synchronous, carcinoma prostate, multiple myeloma

## Abstract

Carcinoma of the prostate is the second most common cancer in males, while multiple myeloma is the 17th most common cancer. The synchronous diagnosis of multiple myeloma and carcinoma of the prostate is a sporadic phenomenon with scarce published literature and a diagnostic and therapeutic dilemma. Here, we present a case of synchronous diagnosis of IgG and lambda subtypes of multiple myeloma with multiple lytic lesions, the revised international staging system (R-ISS 2), and non-metastatic acinar adenocarcinoma prostate, a very high-risk category. The patient received 25 weekly doses of cyclophosphamide, bortezomib, and dexamethasone (CyBorD)-based chemotherapy for myeloma and androgen deprivation therapy with injection leuprolide for prostate cancer. After reasonable disease control, the patient underwent an autologous stem cell transplant for multiple myeloma with melphalan at 140 mg/m2 and was offered definitive radiation therapy for prostate cancer. The potential association between carcinoma of the prostate and multiple myeloma has been hypothesized because of similarities in the tumor microenvironment. There are possible common biological pathways leading to co-stimulatory mechanisms, like interleukin-2 (IL-2), insulin-like growth factor 1 (IGF-1), stromal cell-derived factor 1 (SDF-1), and vascular endothelial growth factor (VEGF). However, they are not proven and warrant further research. This case highlights key areas of diagnosis and management of this sporadic occurrence, along with literature analysis and the need for further research, and is likely to be beneficial for clinicians in decision-making in future cases.

## Introduction

Carcinoma prostate is the second most common cancer and the fifth leading cause of cancer death among men worldwide, with an estimated 1,414,259 cases, amounting to 7.8% of all new cancer cases, 15.1% of cancers in males, and 375,304 deaths in the year 2020 [1]. Prostate cancer is the most common cancer in 112 countries and the leading cause of cancer mortality in 48 countries [2]. The disease burden of prostate cancer is expected to increase due to the aging population and economic growth. Prostate cancer incidence and mortality are very much related to age, with the highest incidence reported in the elderly and the median age at diagnosis being 66 years. African-American men have the highest incidence rates and more aggressive diseases than white men [3].

Screening with serum prostate-specific antigen (PSA) has been adopted in several countries since the 1990s, and a reduction in mortality trend rate has been shown in some countries [4]. There is insufficient evidence from randomized controlled trials, so the recommendation has changed occasionally. In 2012, the United States Preventive Services Task Force (USPSTF) recommended against PSA-based screening, regardless of age. However, in 2018, the USPSTF recommended discussing the potential benefits and harms of screening with their clinician for men aged 55-69 [4].

Multiple myeloma (MM) is the 17th most common cancer, with 98,613 new cases in 2020, or 1.1% of all cases, and the second most common hematologic malignancy [1,2,5]. MM is a neoplasm of clonal plasma cells originating from the post-germinal lymphoid B-cell lineage. It is part of the spectrum of plasma cell dyscrasias, which starts with monoclonal gammopathy of unknown significance (MGUS) and progresses to smoldering myeloma, extramedullary myeloma, MM, and plasma cell leukemia [5]. MM leads to significant morbidity and mortality due to its end-organ damage. It is a disorder of the elderly, and its incidence in the African-American population is twice that of the European-American population. The median age at diagnosis is approximately 66-70 years, with 37% of patients younger than 65 [5].

Synchronous multiple primary cancer (SMPC) is detected together or within six months of another cancer diagnosis, as defined by Warren and Gates's criteria [6]. Accordingly, both neoplasms must be malignant and anatomically separate, and the possibility of metastasis from the index tumor should be excluded. SMPCs likely have a common genetic, hormonal, immunologic, environmental, or iatrogenic link. They are more infrequent than metachronous tumors, defined as more than six months' time interval between the diagnosis [6]. Synchronous diagnosis of carcinoma prostate and multiple myeloma/plasma cell dyscrasia is a sporadic phenomenon scarcely reported in the literature, with less than ten reported cases.

## Case presentation

**Figure 1:**
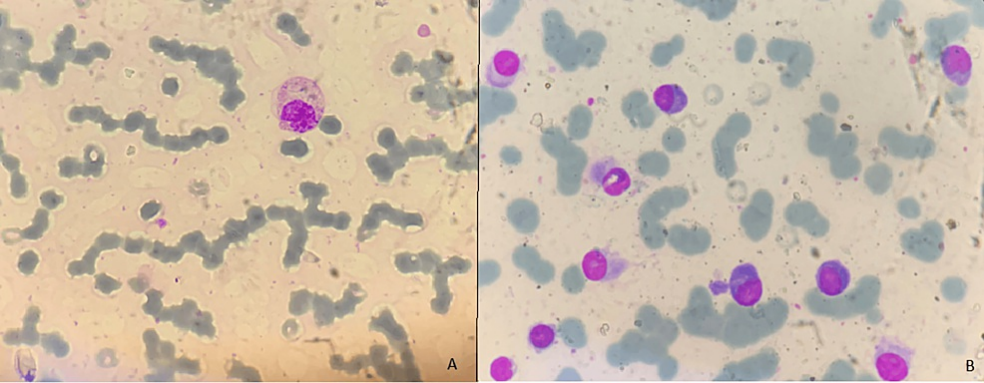
(A) Peripheral smear Leishman staining in ×100 magnification showing plasma cells (blue arrow) with rouleaux formation of erythrocytes (yellow arrow); (B) bone marrow aspiration Leishman ×400 magnification showing plasma cells with round to oval eccentric nucleus with abundant pale blue cytoplasm (red arrow), S/O multiple myeloma.

A 62-year-old male patient with Eastern Cooperative Oncology Group (ECOG) performance status of one presented with complaints of lower back pain radiating to the left lower limb for three months, which was gradually progressive and worsened in the last month. He also had a low-grade fever and significant weight loss of over 10% in the previous six months. Physical examination showed no motor or sensory deficits, and the bilateral planter reflex was the flexor. His hemogram showed a white blood cell (WBC) count of 6.20 × 109/L, a hemoglobin (Hb) level of 6.8 g/dL, and a platelet (PLT) count of 151 × 109/L. Differential counts showed 63% neutrophils (N), 30% lymphocytes (L), 5% monocytes (M), 2% eosinophils (E), and 0% basophils (B). A peripheral smear showed normocytic normochromic anemia with evidence of rouleaux formation (Figure 1A). His erythrocyte sedimentation rate (ESR) was 150 mm at one hour (reference range: 0-14 mm). Other blood investigations showed serum creatinine 3.10 mg/dL (reference range: 0.5-1.5 mg/dL), serum calcium 14.1 mg/dL (reference range: 8.8-10.2 mg/dL), serum total protein 10.9 g/dL (reference range: 6-8 g/dL), serum albumin 2.75 g/dL, and serum lactate dehydrogenase (LDH) 168 U/L (reference range: 135-225 U/L). His bone marrow aspiration showed hypercellular marrow with 68% plasma cells consisting of mature, immature, atypical, binucleate, trinucleate forms, and occasional plasmoblasts (1-2%). Bone marrow aspiration findings favored plasma cell dyscrasia/multiple myeloma (Figure 1B). A bone marrow biopsy showed an excess of plasma cells arranged in clusters and sheets without any evidence of infiltration by carcinoma of the prostate. The bone marrow biopsy images could not be retrieved.

**Figure 2:**
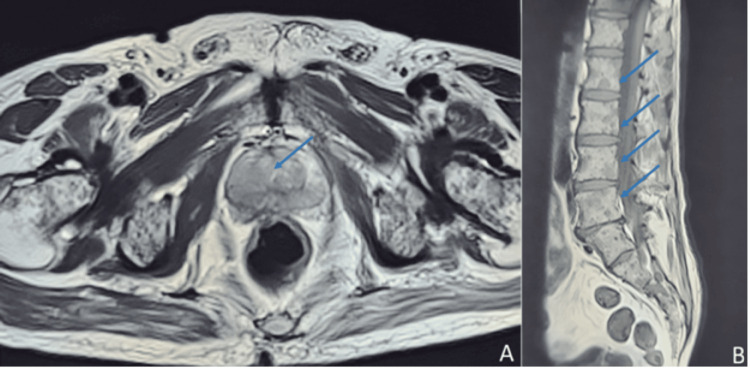
(A) Axial MRI image reveals enlarged prostate gland with evidence of irregular soft tissue lesion involving the peripheral zone on the right side, extending medially crossing the midline with involvement of left side of the prostate (blue arrow); (B) sagittal section MRI of lumbosacral spine reveals multifocal patchy to diffuse areas (blue arrows) of altered signal intensity lesions involving multiple lumbar vertebrae, suggestive of multiple lytic lesions.

Non-contrast magnetic resonance imaging (MRI) for the lumbosacral spine (LS) with whole spine screening and prostate revealed prostatomegaly (49.2 mm × 39.7 mm × 32.2 mm) with a moderate, irregular soft tissue intensity lesion arising from the posterolateral part of the peripheral zone on the right side with evidence of capsular and adjacent neurovascular bundle invasion as well as infiltration of seminal vesicles. The lesion extends to the adjoining transition zone and crosses midline to involve the left posteromedial peripheral zone (Figure 2A). Patchy to diffuse areas of altered signal intensity affected multiple vertebrae in the cervical, dorsal, lumbar, sacral spine, and bilateral iliac bones (Figure 2B). A moderate-sized soft tissue lesion involving the left sacral ala extending anteriorly to involve adjacent soft tissue and sacral neural foramina was noted. The MRI features suggest neoplastic disease and possible metastasis from the primary prostate.

**Figure 3:**
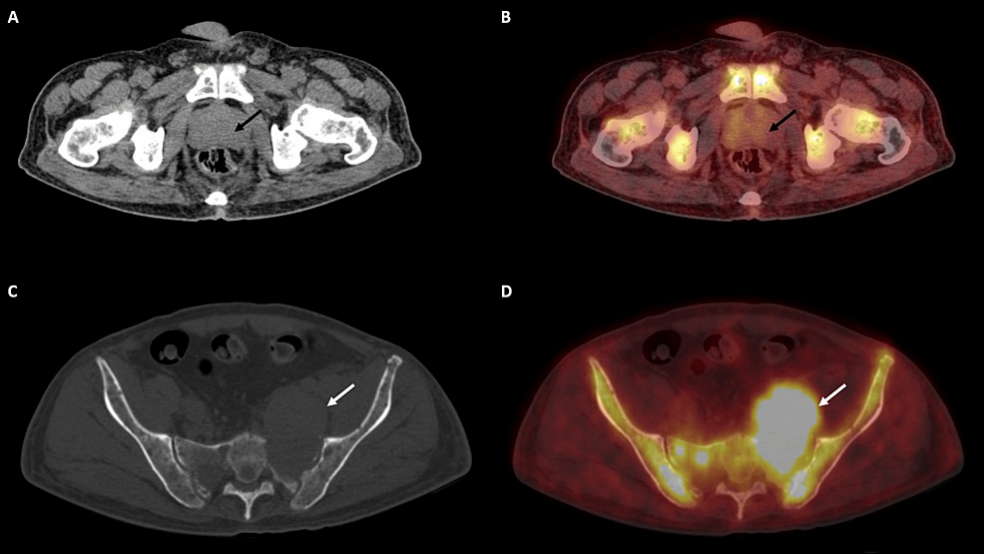
Axial CT image (A) and fused PET CT image (B) reveal an enlarged prostate gland (black arrows) with no abnormal FDG avid lesion. Axial CT image (C) and fused PET CT image (D) reveal a lytic lesion involving the left ala of the sacrum with FDG avid soft tissue component (white arrows). Other FDG avid lytic lesions are also noted in pelvic bones and sacrum. FDG: fluorodeoxyglucose; CT: computerized tomography.

**Figure 4:**
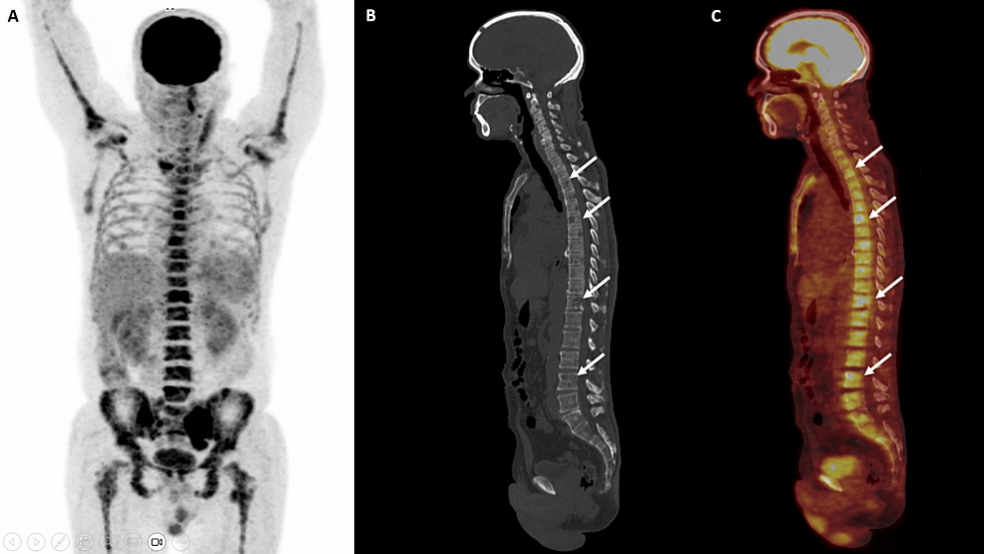
Maximum intensity projection image (A) reveals foci of increased FDG uptake at multiple axial and appendicular skeletal sites. Sagittal CT image (B) and fused PET CT image (C) reveal multiple FDG avid lytic lesions involving the spine (white arrows).

**Figure 5:**
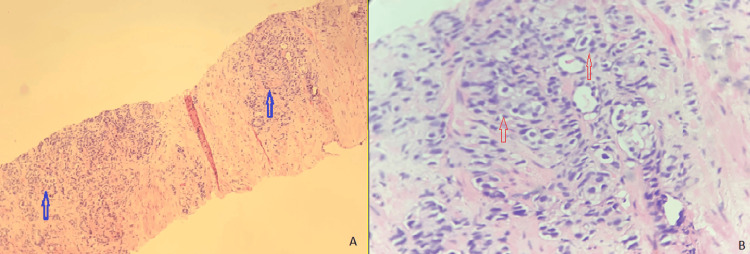
Prostate core biopsy microphotograph showing infiltrative acini in the stroma (blue arrow) (×40, hematoxylin, and eosin); (B) ×400 magnification shows acini lined by malignant cells with nuclear enlargement, nuclear hyperchromasia, and atypia with scant amphophilic cytoplasm (red arrow).

A comprehensive myeloma protein panel revealed distinct bands in the gamma region (band value: 5.20 g/dL), corresponding with IgG and lambda. Multiple myeloma cytogenetic panels by fluorescence in situ hybridization (FISH) on bone marrow aspirate revealed additional signals for the CCND1 gene (chromosome 11), loss of the MAF gene (chromosome 16), and monosomy of chromosome 13. No deletion or translocation of 17p13 (TP53 gene), t(4:14), t(11:14), or t(14:16) was observed. Whole-body positron emission tomography and computed tomography (PET-CT) scans showed multiple fluorodeoxyglucose (FDG) avid lytic lesions scattered in the visualized axial and appendicular skeleton, with evidence of an FDG avid soft tissue component in a lytic lesion involving the left ala of the sacrum measuring approximately 7.2 cm × 4.4 cm, with SUV max 8.3. It favored a differential diagnosis of multiple myeloma (Figures 3-4). Since double primary was our clinical impression, serum PSA was tested, which was 21.52 ng/mL (reference range 0.0-4.0 ng/mL). Transrectal ultrasound (TRUS)-guided prostate biopsy showed invasive prostatic adenocarcinoma, an acinar variant with Gleason's score 4+4, grade group 4 (Figure 5).

The case was discussed in our institutional multidisciplinary board, and a diagnosis of synchronous multiple myeloma, revised international staging system (R-ISS 2), and non-metastatic acinar adenocarcinoma prostate, a very high-risk category, was made. The patient received systemic therapy for multiple myeloma with 16 weekly doses of bortezomib in a dose of 1.3 mg/m^2^, cyclophosphamide in a dose of 300 mg/m^2^, and dexamethasone in a dose of 40 mg (CyBorD). Because of the high serum creatinine of 3.10 mg/dL at diagnosis, monthly zoledronic acid was added from week eight of CyBorD after normalization of serum creatinine. He received two doses of a gonadotropin-releasing hormone (GnRH) agonist, leuprolide (22.5 mg), at three-month intervals for prostate carcinoma. Follow-up serum PSA was 1.17 ng/mL at three months of initiation of leuprolide. Follow-up multiple myeloma monitoring panel at four months of initiation of CyBorD showed M spike 0.50 g/dL, serum beta 2 microglobulin 2888 ng/ml, serum kappa free light chain 20.47 mg/L, serum lambda free light chain 16.22 mg/L, and serum Kappa lambda ratio 1.262. The baseline and follow-up parameters are shown in Table 1.

**Table 1:** Multiple myeloma along with serum PSA and kidney function parameters at baseline and on follow-up. M protein: myeloma protein; PSA: prostate-specific antigen.

Investigation	At diagnosis	On follow-up	Reference range
Serum immunoglobulin A (IgA)	31 mg/dL	71.50 mg/dL	40–350 mg/dL
Serum immunoglobulin G (IgG)	7830 mg/dL	1027 mg/dL	650–1600 mg/dL
Serum immunoglobulin M (IgM)	59 mg/dL	41.60 mg/dL	50–300 mg/dL
serum Kappa-free light chain	−19.70 mg/L	20.47 mg/L	3.30–19.40 mg/L
serum lambda-free light chain	667.00 mg/L	16.22 mg/L	5.71–26.30mg/L
serum Kappa lambda ratio	0.030	1.262	0.26–1.65
serum beta 2 macroglobulin	16,619 ng/mL	2888 ng/mL	609–2366 ng/mL
M spike	5.2 g/dL	0.50 g/dL	-
Hemoglobin	6.8 g/dL	11.0 g/dL	11.5–16.5 mg/dL
Serum creatinine	3.10 mg/dL	1.42 mg/dL	0.5–1.5 mg/dL
Serum PSA	28.100 ng/mL	1.17 ng/mL	0.0–4.0 ng/mL

After 25 weeks of CyBorD treatment, the patient underwent autologous stem cell transplantation. His pre-stem cell transplant workup showed cellular marrow (varying from 05% to 30%) with no excess of plasma cells, and measurable residual disease (MRD) on bone marrow aspirate revealed approximately 0.08% plasma cells gated in the bright CD38 and CD138 regions. Peripheral blood stem cell apheresis was collected, and 225 ml of stem cell product with a CD34 cell dose of 5.8 million/kg had 99% viability. Conditioning was done with melphalan at 140 mg/m^2^ on day −1. A stem cell product with a CD34 cell dose of 5.8 million/kg was infused on day zero. The patient developed grade two diarrhea and mucositis, which was managed symptomatically. Nadir TLC count of 100 cells/µL reached on day +5, increased to 810/µL on day +9 (neutrophil engraftment). Platelet count increased to 37,000 (platelet engraftment) on day +10. His follow-up bone marrow examination showed cellular bone marrow with no excess plasma cells.

MRD on bone marrow aspirate revealed CD38 and CD138-positive abnormal plasma cells comprising 0.0018% of total nucleated cells. The patient is on three monthly leuprolides and has been planned for maintenance of bortezomib and radical intent radiation therapy for prostate cancer after three months.

## Discussion

Secondaries to bone are the most common type of cancer affecting the bones. Bone metastases are divided into osteoblastic, osteolytic, or mixed, depending on their appearance on imaging and the histological type of cancer [7]. Radiographic lucency is the hallmark of osteolytic lesions usually seen in multiple myeloma, thyroid cancer, and melanoma, in which osteoclast-mediated bone destruction occurs. Dense and sclerotic lesions on radiology characterize osteoblastic lesions and are usually reported in carcinoma of the prostate, small cell lung cancer, Hodgkin's lymphoma, and carcinoid tumors. Mixed lesions show osteolytic and osteoblastic features, usually seen in gastrointestinal tumors or squamous cell carcinomas [7].

Synchronous malignancies usually have a common genetic, infectious, environmental, or occupational association, but few are coincidental. Limited literature exists about synchronous or metachronous plasma cell dyscrasias and prostate cancers (Table 2) [8-21]. Despite an extensive search, we could find six reported cases of myeloma, four cases of metachronous diagnosis of myeloma, two cases of MGUS, two cases of smoldering myeloma, and one case of plasmacytoma with prostate carcinoma.

**Table 2:** Details of previously published case reports of carcinoma prostate and plasma cell dyscrasias. MGUS: monoclonal gammopathy of unknown significance; NA: not available; USA: United States of America.

Author	Country	Year	Topic	Age	Myeloma details	Prostate cancer details
Huang et al. [8]	USA	2002	Recurrence of prostate adenocarcinoma presenting with multiple myeloma simulating skeletal metastases of prostate adenocarcinoma	77	Metachronous myeloma after eight years	Non-metastatic
Lopez et al. [9]	Spain	2007	Prostate adenocarcinoma and synchronous multiple myeloma: a case report	63	Smoldering myeloma	Metastatic HSPC
Yoshinaga et al. [10]	Japan	2010	Multiple myeloma diagnosed during hormonal therapy for prostate cancer: report of two cases	73, 70	NA, metachronous development	Metastatic HSPC
Eiko Klimanta et al. [11]	USA	2011	Synchronous presentation of a primary iliac lymph node plasmacytoma and a prostate adenocarcinoma	68	Left iliac lymph nodal chain plasmacytoma	Metastatic HSPC
Nae Yu Kim et al. [12]	Korea	2011	Multiple myeloma with bi-clonal gammopathy accompanied by prostate cancer	58	IgG Kappa and IgA Lambda	Metastatic HSPC
Mirna Sucic et al. [13]	Croatia	2012	A patient with prostate cancer and multiple myeloma—diagnostics and possible association of both diseases	63	NA	Non-metastatic
Tushar Sehgal et al. [14]	India	2014	Synchronous Occurrence of Prostate Carcinoma and Multiple Myeloma: A Case Report	62	IgA Lambda	Metastatic HSPC
Pankaj Mathur et al. [15]	USA	2017	Metastatic prostate cancer with bone marrow infiltration mimicking multiple myeloma	85	Smoldering myeloma	Metastatic HSPC
Adrianzen et al. [16]	Italy	2018	Synchronous bone metastasis from multiple myeloma and prostate adenocarcinoma as initial presentation of coexistent malignancies	71	IgA Kappa	Metastatic HSPC
Pramanik et al. [17]	India	2018	Monoclonal gammopathy in prostate carcinoma: a case report and review of literature	65	MGUS	Metastatic HSPC
Waqas et al. [18]	USA	2018	Prostate cancer leading to monoclonal gammopathy of undetermined significance: a case report	76	MGUS	Metastatic HSPC
Yelda Vyas et al. [19]	India	2018	Coexisting prostate adenocarcinoma with multiple myeloma: a rare case report	83	light chain multiple myeloma	Metastatic HSPC
Dass et al. [20]	India	2020	Myeloma co‑existing with prostatic carcinoma: clues from a “non‑coagulable” prothrombin time	70	IgA Lambda	Metastatic HSPC
Abolghasem et al. [21]	Iran	2023	Prostate cancer, chronic myelogenous leukemia and multiple myeloma in a single patient: a case report and review of the literature	85	IgG Kappa, metachronous development in 2023	Metastatic HSPC (2016), CML (2017)

The potential association between carcinoma of the prostate and multiple myeloma has been hypothesized because of similarities in the tumor microenvironment of these cancers. There are possible common biological pathways leading to co-stimulatory mechanisms, but they are not proven and warrant further research. The critical growth factors and antiapoptotic cytokines are interleukin-6 (IL-6) and insulin-like growth factor 1 (IGF-1), which are released by myeloma cells and have a role in prostate cancer development by activation of the Ras/Raf/MAPK pathway or stromal-derived factor 1 (SDF-1) [22,23]. The relative risk of prostate cancer was 4.3 in the group with the highest quartile of plasma IGF-1, as compared to the lowest quartile, as per a study by Chan et al. [24]. SDF-1 is a common chemokine that causes selective adhesion of myeloma cells to bone tissue and participates in the bone metastasis of prostate cancer. C-myc dysregulations are found in 45% to 90% of multiple myeloma cases, which correlates with a higher grade of malignancy [23]. C-myc amplification is also associated with poorer survival in prostate cancer patients. Mutation of the IL-6 gene on chromosome 7p2 has been detected in prostate cancer and MM in the review of genetic profiles of cancers [25]. IL-6 is also associated with other cancers like breast, renal cell, and ovarian cancers, Hodgkin lymphomas, and T-cell lymphomas. IL-6 is considered the principal growth factor in the progression of plasma cell disorders [23,25].

Prostate cancer is initially dependent on androgens for proliferation, called hormone-sensitive prostate cancer (HSPC), which subsequently progresses to an androgen-independent state known as castration-resistant prostate cancer (CRPC) [25]. There is evidence that IL-6 levels correlate with prostate cancer tumor burden, and it contributes to the progression of the disease by inducing the activation of the androgen receptor in the absence of androgen, promoting neuroendocrine differentiation and vascular endothelial growth factor (VEGF) expression [25]. Anti-IL-6 antibodies have shown sensitization of resistant prostate cancer cells to chemotherapy in vitro. IL-6 is being considered a potential target for the treatment of prostate cancer [26]. The immunocompromised state in myeloma patients is a possible factor that can contribute to more aggressive disease biology and the rapid progression of prostate cancer [23]. Analysis of the cancer registry has shown some common genetic variations, leading to an increased risk of multiple myeloma in families with many prostate cancers, especially those diagnosed early [23].

There are no defined guidelines regarding the treatment of synchronous malignancies. The treatment sequence and modalities are decided based on the stage of diseases, symptoms, aggressiveness of diseases, co-morbidities, expected survival, available treatment options, interaction among treatment modalities, performance status of patients, etc. In the current case, the patient was symptomatic for multiple myeloma while having minimal symptoms of prostate cancer with mild urinary obstructive symptoms. After our institutional multidisciplinary discussion, treatment of multiple myeloma was started with the CyBorD regime with the intention to go for autologous stem cell transplantation after getting a response and reasonable disease control. Induction chemotherapy followed by autologous stem cell transplant in transplant-eligible cases is the standard of care for managing MM [5,10-16]. Androgen deprivation therapy with an injection of leuprolide at three-month intervals was started for prostate carcinoma with a plan to go for definitive radiation therapy in the future, which is the standard of care in locally advanced prostate cancer cases [3,4,10-16]. Since there is no significant interaction between the planned myeloma treatment and leuprolide, this treatment was very well tolerated, and the patient responded well to treatment for both multiple myeloma and prostate cancer.

The etiology of lytic bone lesions in our case was MM. Carcinoma of the prostate can also have lytic bone metastasis in some cases. In a retrospective analysis of 119 cases with vertebral metastasis in carcinoma prostate, lytic metastasis was seen in 19%, and a mixed pattern of metastasis was seen in 26% of the cases [27]. There was a possibility of missing the diagnosis of MM in our case if the myeloma profile and bone marrow aspiration were not done at baseline. Rarely, prostate cancer and myeloma cell deposits can be seen together in bone marrow [16]. A detailed and careful bone marrow examination and immunohistochemistry are warranted if there is suspicion of synchronous deposits in bone marrow.

## Conclusions

Synchronous multiple myeloma and prostate cancer are extremely rare and possess a unique diagnostic and therapeutic challenge. A bone marrow examination is prudent whenever clinical suspicion of another associated pathology is present. This case highlights the importance of strong clinical acumen and the role of multidisciplinary discussions in managing these sporadic cases. The sequencing of treatment modalities, interaction among treatment options, patient's performance status, organ functions, and other factors guide the treatment of this complex disease. CyBorD is an effective regime for myeloma, especially for patients with baseline renal dysfunction, followed by autologous stem cell transplantation, which is the standard of care. This case has summarized all relevant published literature in one place and will likely help clinicians in their clinical decision-making and serve as a reference for future cases. This case also highlights the need for future research to prove the association hypothesis between these cancers.
